# A new method for non‐invasive determination of effective pulmonary blood flow and cardiac output in spontaneously breathing subjects

**DOI:** 10.1113/EP093079

**Published:** 2025-09-04

**Authors:** Andras Gedeon, Jakob Jansson, David Patrickson, Mats Wallin

**Affiliations:** ^1^ Mincor AB Stockholm Sweden; ^2^ Spirotronic AB Stockholm Sweden; ^3^ eHeart AB Stockholm Sweden; ^4^ Department of Physiology and Pharmacology Karolinska Institutet Stockholm Sweden

**Keywords:** cardiac output, non‐invasive, pulmonary blood flow, rebreathing, spontaneous breathing

## Abstract

The differential Fick method is well established for measuring effective pulmonary blood flow (EPBF) and cardiac output (CO) but until now it has only been used for patients on mechanical ventilation. Here we present and evaluate a new approach adapted to spontaneous breathing situations. Ten healthy subjects with diverse anthropometric and respiratory parameters were studied in the sitting position. Rebreathing through a dead space of precisely known volume and recording the resulting rise in the end‐tidal CO_2_ value allowed the determination of EPBF. The shunted blood flow fraction was estimated from the arterial oxygen saturation to obtain cardiac output (FickCO). Two measurements were made on each subject 15 min apart. Reference values for cardiac output (RefCO), were calculated as the product of stroke volume and heart rate where the stroke volume was measured with established echocardiography techniques. Heart rate and arterial oxygen saturation were measured with an ordinary pulse oximeter. Comparing FickCO to RefCO using a Bland–Altman analysis, we obtained a mean bias of 0.03 L/min, limits of agreement (LoA) of +1.43 to –1.37 (95% CI) L/min and a percentage error (PE) of 0.25. For the mean of two FickCO observations, we obtained a mean bias of −0.04 L/min, LoA +0.94 to −1.01 (95% CI) and PE of 0.17. The differential Fick method can be adapted to spontaneously breathing situations with good absolute accuracy using simple equipment. Short data collection times make it possible to use the mean of repeated observations and thereby get adequate precision. The new method could therefore be of value both in the pre‐operative and the post‐operative setting.

## Abstract (highlights)

- **What is the central question of this study?**
  Can the differential Fick method for measuring effective pulmonary blood flow be adapted for use in spontaneously breathing subjects and if so, how?
- **What is the main finding and its importance?**
  The differential Fick method can be adapted for measuring effective pulmonary blood flow using simple inexpensive equipment and the method can be validated with echocardiography as reference. The importance of this finding is that being a non‐invasive procedure, it can be used whenever measurement of pulmonary blood flow is of interest, as for instance in pre‐operative and post‐operative settings and possibly also in more resource limited out‐of‐hospital environments.

## 1 INTRODUCTION

The differential Fick method is well established for the measurement of non‐shunted effective pulmonary blood flow (EPBF) (Dueck, 2004; Gama de Abreu et al., 1997; Gedeon et al., 1980; Jaffe, 1999). The method utilizes the fact that due to the large body stores of carbon dioxide and venous recirculation time, a suddenly induced change in the CO_2_ elimination (${\dot{V}}_{CO_{2}}$) from the lungs will immediately change the arterial blood content while the mixed venous blood content will remain unchanged for a certain period. During this time interval the change in ${\dot{V}}_{CO_{2}}$ will be proportional to the change in the arterial CO_2_ partial pressure, which in turn can be approximated with the change in the end tidal CO_2_ ($P_{\mathrm{ETC}O_{2}}$). The procedure is non‐invasive, and the constant of proportionality determines EPBF.

There are several ways to change ${\dot{V}}_{CO_{2}}$, and it is most conveniently done when the patient is on a mechanical ventilator. Therefore, until now, the method has been used only for ventilated patients in the intensive care unit or operating room. Initial work (Gedeon et al., 1980) as well as the most recent developments (Hällsjö Sander et al., 2014; Karlsson & Lönnqvist, 2022; Sigmundsson, 2019) have used manipulations of the breathing pattern of the patient. However, since the first introduction of the CO_2_ rebreathing approach (Gedeon, 1985), this way of changing ${\dot{V}}_{CO_{2}}$ has been well studied (Gama de Abreu et al., 1997; Haryadi et al., 2000; Jaffe, 1999; Gedeon et al., 1992) and also the method chosen for a commercial implementation (Jaffe, 1999).

In the present work we adopt the rebreathing method and extend its use to measure EPBF also in spontaneously breathing subjects and we evaluate how well the system performs relative to cardiac output measurements made by standard echocardiography.

## 2 METHODS

### 2.1 Ethical approval

This study has been approved by the Swedish Ethical Review Authority (15 April 2025, No. 2025‐01936‐02). The decision was based on judgement from legal and medical professionals. This approval assures conformity with the latest version of the *Declaration of Helsinki* and with other ethical requirements including those on written informed consent and consent to publish as well as data handling and storage procedures.

### 2.2 Physical and respiratory characteristics

Ten subjects, four women and six men, with no known cardio‐pulmonary disease, were recruited and studied in the sitting position. They were between 39 and 70 (mean 55) years old, with weights between 62 and 99 (mean 80) kg, tidal volume (TV) was between 0.58 and 1.70 (mean 1.1) L and respiratory rate (RR) between 4.6 and 16.8 (mean 11) breath/min. For women mean RR and TV were 8.9 breath/min and 1.2 L, and for men, 9.7 breath/min and 1.1 L.

### 2.3 Description of the equipment and the procedure

**FIGURE 1:**
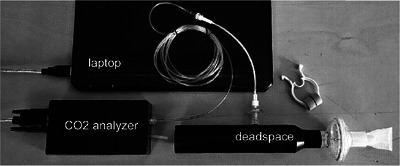
The equipment used for this study.

The equipment used for this study is shown in Figure 1. It was made up of a nose clip, a dead space volume (DV), a sampling IR CO_2_ analyser (ET600 Side stream CO_2_ CGM Module; Ronseda Electronics Co., Ltd, Shenzen, China) and a laptop computer for data collection. TV was measured for each subject with a Cosmed Quark PFT unit (Cosmed SRl, Rome, Italy).

The dead space volume was formed by connecting a mouthpiece, a respiratory filter (ZF‐007 Shandong Zhenfu Medical Device Co., Ltd, Rizhao, China) and a cylindrical pipe. The combined volume was determined with an accuracy of better than 3 mL. The sampling port of the analyser was connected to the pipe through a Nafion tube and a sampling filter. The sampling point was −10 mm inside the lumen of the pipe. Three different dead space volumes were used: 0.168, 0.238 and 0.306 L. They were chosen dependent on the known tidal volume of the subject so that the ratio DV/TV was in the range 0.2–0.3.

The subjects were instructed to sit still for about 5 min. During this time a pulse oximeter (Braun Pulse Oximeter 1, Xuzhou, China) was applied to a finger and the heart rate (HR) and the oxygen saturation $S_{aO_{2}}$ were noted. We observed $S_{aO_{2}}$ in the range 93–99% (mean 96%) and HR in the range 60–86 (mean 73) beats/min. The subjects were then asked to apply the nose clip and to start breathing in the dead space, beginning with an expiration and to breathe in a normal manner until instructed to stop. Data collection took typically 30 s and always less than 50 s. After sitting still for about 15 min, this procedure was repeated for a second set of measurements.

Reference cardiac output (RefCO) was measured in the sitting position with a GE Vivid E95 ultrasound unit using the velocity–time integral method. Here the stroke volume is obtained from the approximately circular area of outflow from the left ventricle, times the outflow velocity of the blood. As a validation check, the stroke volume was also calculated from the 3D reconstruction of the left ventricle as the difference between the volumes pre and post systole. The two methods agreed within about 3% in all cases. Stroke volume and FickCO were measured by separate persons at separate locations and the results were blinded to each other. Stroke volume was measured between the two FickCO measurements.

According to the differential Fick method (Gedeon et al., 1980), EPBF can be obtained from the expression:

$$\mathrm{EPBF}=\left(1/S\right)\times \left(\mathrm{delta}\;{\dot{V}}_{CO_{2}}\right)/\mathrm{delta}\;P_{\mathrm{ETC}O_{2}}$$

where *S* is the slope of the CO_2_ dissociation curve, delta ${\dot{V}}_{CO_{2}}$ is the reduction in CO_2_ outflow from the lungs due to rebreathing and delta $P_{\mathrm{ETC}O_{2}}$ is the resulting increase in the end‐tidal $P_{\mathrm{ETC}O_{2}}$ value.

In our approach (Gedeon, 2023), delta ${\dot{V}}_{CO_{2}}$ is obtained by first determining the volume of CO_2_ within the dead space at the end of expiration and then by multiplying this value with the mean RR during rebreathing. The CO_2_ volume in the dead space is calculated as the product of the known dead space volume and a representative sample of the CO_2_ partial pressure within the dead space at the end of expiration.

With DV/TV < 0.3, the CO_2_ partial pressure will decrease slowly and linearly within the dead space away from the subject. A sampling point that divides the dead space into two equal parts will therefore provide a representative average value for the entire dead space, av $P_{\mathrm{ETC}O_{2}}$. We thus get:

$$\;\mathrm{delta}\;{\dot{V}}_{CO_{2}}=\mathrm{av}\;P_{\mathrm{ETC}O_{2}}\times \mathrm{DV}\times \mathrm{mean}\mathrm{RR}$$

We use Capek's formula for *S* (Capek & Roy, 1988):

$$S=\left(1.34\times \mathrm{Hb}+18.34\right)/\left(1+0.193\times P\right)$$

with hemoglobin value (Hb) = 13.8 for women and Hb = 15.0 for men. In accordance with a previous analysis (Gedeon et al., 1980, 1992) for healthy subjects, the slope can be evaluated at a CO_2_ partial pressure *P* = $P_{\mathrm{ETC}O_{2}}$ + 10 mmHg where $P_{\mathrm{ETC}O_{2}}$ is the value measured at the start of rebreathing.

Shunted blood flow, passing where the ventilation/perfusion ratio in the lung is small or zero, can be estimated from $S_{aO_{2}}$ values based on the work of Nunn (1993). His results have been given a graphical presentation (Jaffe, 1998) and from this an analytical expression can be found for the shunt fraction (Sh) as a function of $S_{aO_{2}}$. For breathing room air, we get:

$$\mathrm{Sh}=0.0154\times \surd 25.3\times \left(100-S_{aO_{2}}\right)+{\left(100-S_{aO_{2}}\right)}^{2}$$

The cardiac output from our measurements, FickCO, to be compared to the reference, RefCO, is then given by:

FickCO=EPBF/(1−Sh)

### 2.4 Statistics

All statistical calculations were done with the MedCalc statistical software from MedCalc Software Ltd (Ostend, Belgium). To show criterion validity for the new method we used the Bland–Altman procedure when comparing to the reference and Student's paired *t*‐test for comparing the two sets of FickCO measurements and an unpaired *t*‐test for assessing how women and men compare to reference.

## 3 RESULTS

**FIGURE 2:**
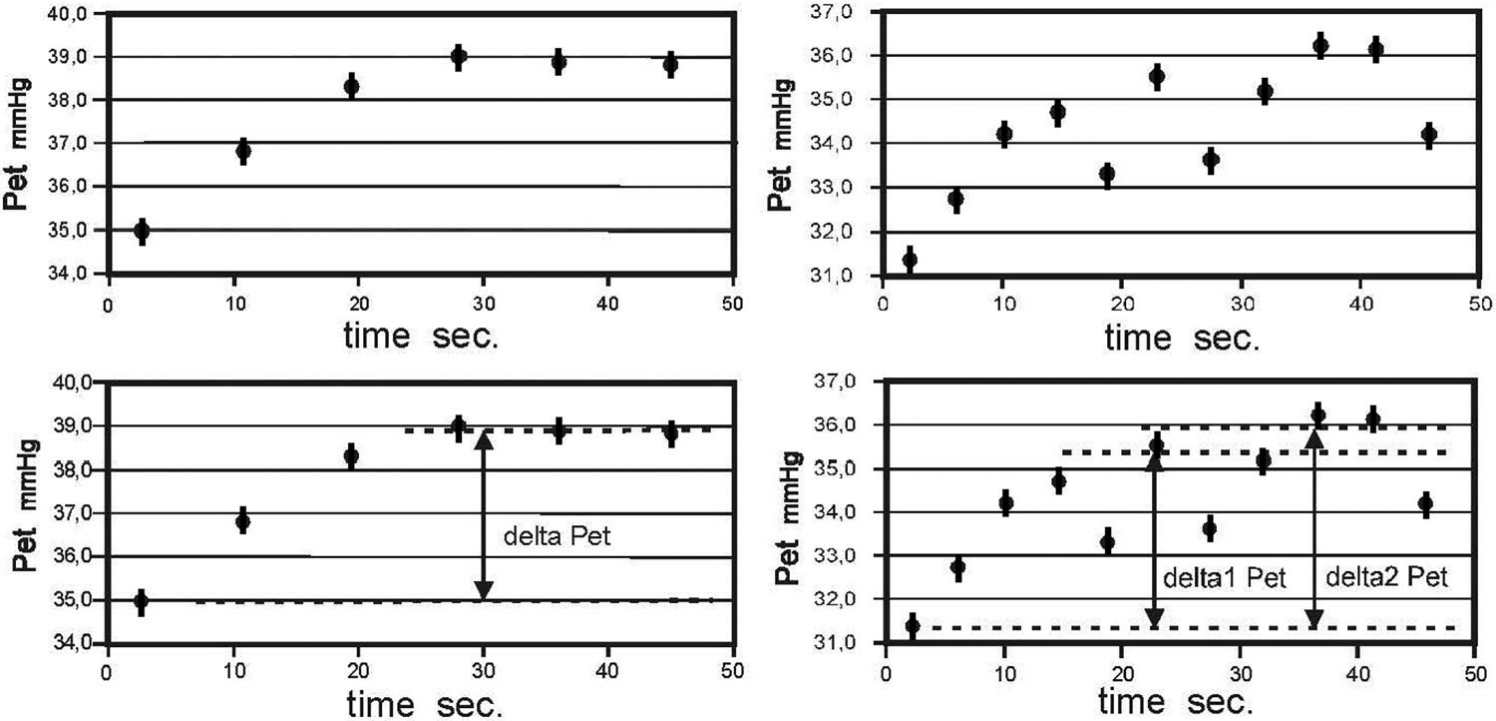
Two cases of raising $P_{\mathrm{ETC}O_{2}}$ due to rebreathing.

Figure 2 shows two cases of raising $P_{\mathrm{ETC}O_{2}}$ due to rebreathing. On the left is a nearly perfect data set and on the right is a more typical recording. The left recording produces a well‐defined delta $P_{\mathrm{ETC}O_{2}}$ with the result FickCO = 6.1 L/min to be compared with RefCO = 5.9 L/min. The recording on the right opens the way for alternative analyses. Two different possible delta $P_{\mathrm{ETC}O_{2}}$ values are shown. They correspond to FickCO = 5.2 L/min and FickCO = 5.7 L/min, that is an uncertainty of 0.5 L/min or about 10%. In this case RefCO = 5.3 L/min indicating that delta2 $P_{\mathrm{ETC}O_{2}}$ is the more correct interpretation.

We were unable to evaluate four registrations in the first set of measurements and three in the second due to irregular breathing and/or leakage around the mouthpiece. We thus obtained *n* = 13 data points for the comparison to RefCO.

**FIGURE 3:**
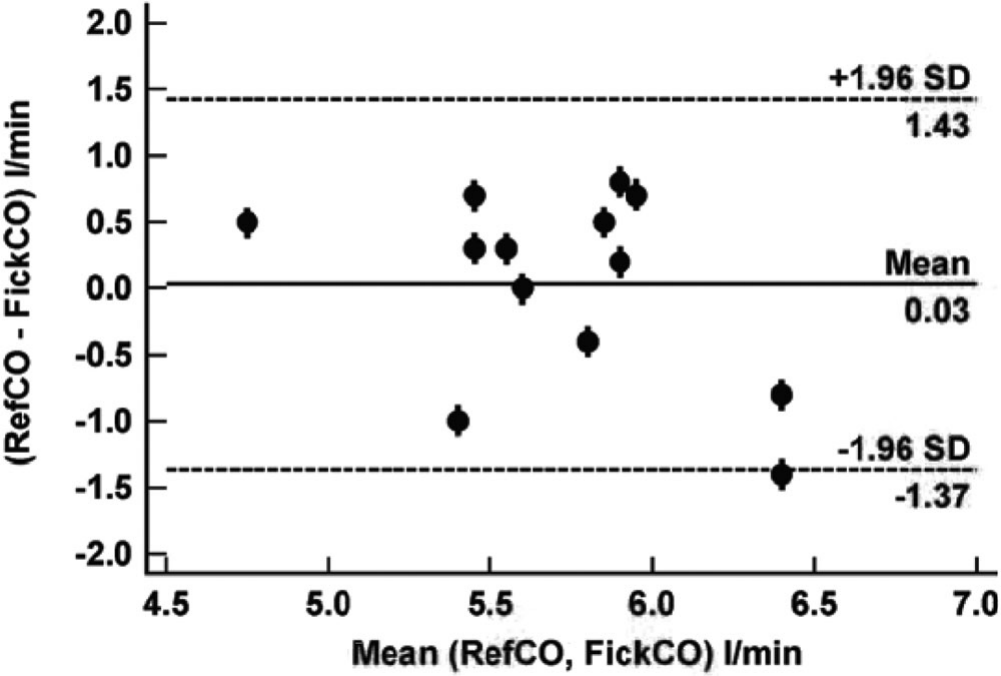
Bland–Altman presentation for REfCO versus FickCO data.

The result is shown in Figure 3 used the Bland–Altman presentation (Bland & Altman, 1986) for RefCO versus FickCO data. We obtained a mean bias of 0.03 L/min with limits of agreement (LoA) +1.43 to −1.37 (95% CI) L/min and a percentage error (PE) of 0.25. An unpaired *t*‐test comparing RefCO–FickCO for women and men showed a mean difference of −0.083 L/min with SD of 0.71 L/min and LoA of −1.00 to +0.83 (95%CI) L/min with *t* = −0.20 and two‐tailed probability of 84.4%.

**FIGURE 4:**
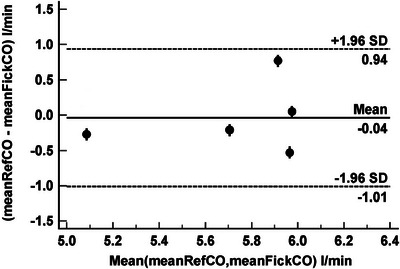
Bland–Altman presentation comparing the mean of two FickCO values to the mean RefCO value.

Figure 4 shows the Bland–Altman presentation comparing, for each subject (*n* = 5), the mean of two FickCO values to the mean RefCO value. We obtained mean bias = −0.04 L/min, LoA +0.94 to −1.01 (95% CI) L/min and PE = 0.17.

A paired *t*‐test for the two set of FickCO measurements showed mean difference = 0.038 L/min with SD = 0.50 L/min, LoA +0.65 to −0.58 (95% CI) L/min with *t* = 0.17 and a two‐tailed probability of 87.2%.

## 4 DISCUSSION

For spontaneous breathing situations, rebreathing is a natural choice for altering ${\dot{V}}_{CO_{2}}$. It perturbs the alveolar ventilation without involving cooperation from the subject and allows for a very simple measuring system that is in addition highly fault tolerant (Gedeon, 1985, 2023). The expression for EPBF is a ratio of two differences and since all data are collected with a single analyser the result will be insensitive both to instrument off‐set and to the gain setting.

Furthermore, by obtaining ${\dot{V}}_{CO_{2}}$ using precise volume measurements and not as is usual from integrated flow and concentration versus time data, our approach offers both simplicity and better measuring accuracy that other rebreathing techniques mostly lack (Peyton & Thomson, 2004). When comparing our method to the reference method, the exchangeability criteria of Critchley and Critchley (1999) are fulfilled both with a single observation (PE = 0.25) and when the average of two observations is considered (PE = 0.17).

The precisely known dead space volume, the key component in our method, should be chosen so as not be too small compared to the tidal volume because this would produce smaller increases in $P_{\mathrm{ETC}O_{2}}$ and thus reduce useful information and precision. It should not be chosen as too large because this might lead to a non‐linear concentration gradient within the dead space so that the $P_{\mathrm{ETC}O_{2}}$ value would no longer be representative of the mean concentration in the entire volume. We have found that a suitable interval for the ratio DV/TV is 0.2–0.3.

The slope of the CO_2_ dissociation curve (*S*) changes slowly and although it depends on parameters that are not exactly known, they can still be estimated well enough not to give rise to significant errors.

As an example, the CO_2_ partial pressure where the slope of the CO_2_ dissociation curve (S) is to be determined depends on the end‐tidal to arterial $P_{CO_{2}}$ difference. In our healthy subjects this is typically on average 4 mmHg (McSwain et al., 2010). A change of 2 mmHg in this value would change FickCO about 5%. Also, if Hb in the expression for *S* was to change 10%, this would also change FickCO about 5% (as also stated in Capek & Roy, 1988). According to Whitehead et al. (2019), Hb analysers, including handheld devices used in resource limited situations, measure better than ±7% compared to the gold standard. Therefore, whenever deemed necessary a measured Hb value can be used in the expression of *S* and will introduce less than 5% error in the value of EPBF.

The shunt fraction calculation used to obtain FickCO from EPBF introduces both absolute error and variability. A typical error of ±2% in the value of $S_{aO_{2}}$ translates into an error in FickCO of about ±9% when $S_{aO_{2}}$ = 98% while the same error in $S_{aO_{2}}$ translates into an error of only about ±5.5% when $S_{aO_{2}}$ = 96%.

Our test group was recruited to represent a rather wide range of physical and respiratory parameters; however, as a group, women and men were very well matched and our data show no significant statistical difference between the groups as regards agreement with the reference values.

The most challenging aspect of the new method contributing to the variability in the data is irregular breathing. Figure 2 shows both a nearly ideal registration and a typical one. However, we also made seven measurements that could not be evaluated.

About half of these were due to known or suspected leakage. Two of the subjects had difficulty in maintaining a tight seal around the mouthpiece. Leakage ruins the measurement entirely because in this situation rebreathing will not produce a consistent increase in $P_{\mathrm{ETC}O_{2}}$. Another subject had strongly irregular breathing at the first measurement but performed much better the second time, showing that the learning curve of following even a simple procedure differs and must be taken into account. As apparent from Figure 2, a significant amount of irregular breathing can be tolerated if it impacts the latter part of the rebreathing period. However, if it also occurs in the very beginning, it will make the analysis of the data impossible. This situation we encountered in two of the seven cases.

Therefore, it may be advisable to abort a measurement if the first two to three breaths of the rebreathing period do not produce a reasonably smooth continuous rise in $P_{\mathrm{ETC}O_{2}}$. In doing so one can both avoid completing a test that will be very difficult if not impossible to evaluate and at the same time allow a repeated attempt at shorter notice.

Finding ways to promote regular breathing is clearly of significant value but precision can be improved by repeating the measurement and using average values instead of single observations. Figures 3 and 4 show this when averaging just two values. In the present study we have chosen to repeat the measurement once, after 15 min, to ascertain that the gas exchange changes introduced by the first measurement had no influence on the second and, as expected, no statistical difference was found between the two sets of measurements.

However, there are good reasons to believe that with a typical rebreathing time of only about 30 s the measurement could have been repeated faster, possibly as often as every 5 min (Capek & Roy, 1988).

### 4.1 Conclusions

The differential Fick method can be adapted to spontaneous breathing situations using simple equipment. The absolute accuracy of FickCO compares favourably with the clinically accepted reference. Short measuring times allow for using an average of repeated measurements assuring satisfactory precision. The method could potentially have considerable value in both pre‐operative and post‐operative settings. Attention should be given to setting up test conditions and procedures that favour regular breathing.

## AUTHOR CONTRIBUTIONS

Andras Gedeon: Conception, design, data analysis and presentation. Jakob Jansson: Technical development and data collection. David Patrickson: Methodology for and measurement of the reference data. Mats Wallin: Expertise in the Fick method, study design and presentation. All authors have read and approved the final version of this manuscript and agree to be accountable for all aspects of the work in ensuring that questions related to the accuracy or integrity of any part of the work are appropriately investigated and resolved. All persons designated as authors qualify for authorship, and all those who qualify for authorship are listed.

## CONFLICT OF INTEREST

A.G. is a shareholder and employee of Mincor AB. The other authors have no competing interests, financial or non‐financial, to disclose.

## Data Availability

All data supporting the results are in the paper. All data from this project are stored on two laptops at different locations as approved by the Swedish Ethical Review Authority and can be accessed as Supporting Information by contacting the corresponding author. Ethical and legal requirements as regards identification of the source of the data has been and will be followed.
